# A Statistical Explanation of the Dunning–Kruger Effect

**DOI:** 10.3389/fpsyg.2022.840180

**Published:** 2022-03-25

**Authors:** Jan R. Magnus, Anatoly A. Peresetsky

**Affiliations:** ^1^Department of Econometrics and Data Science, Vrije Universiteit Amsterdam, Tinbergen Institute, Amsterdam, Netherlands; ^2^Department of Applied Economics, Faculty of Economic Sciences, Higher School of Economics (HSE University), Moscow, Russia

**Keywords:** boundary conditions, tobit model, Dunning–Kruger effect, conditional expectation, underestimation and overestimation

## Abstract

An explanation of the Dunning–Kruger effect is provided which does not require any psychological explanation, because it is derived as a statistical artifact. This is achieved by specifying a simple statistical model which explicitly takes the (random) boundary constraints into account. The model fits the data almost perfectly.

**JEL Classification:**

A22; C24; C91; D84; D91; I21

## Introduction

The Dunning–Kruger (DK) effect states that people with low ability tend to overestimate their ability. This hypothetical cognitive bias was first described in Kruger and Dunning (1999) and, if true, it is potentially important and dangerous, because it means that people of low ability not only perform tasks poorly but (even worse) that they think that they perform these tasks well. Dunning and Kruger claim that the reason for this bias is that people of low ability are not good in seeing and judging themselves (a deficit in metacognitive skills). A closely related effect, also important but arguably less dangerous, is that people of high ability tend to underestimate their ability. This second effect, although not discussed in Kruger and Dunning (1999), is often also associated with their names. The DK effect and Dunning and Kruger's explanation of it has been discussed and challenged extensively.

**Figure 1:**
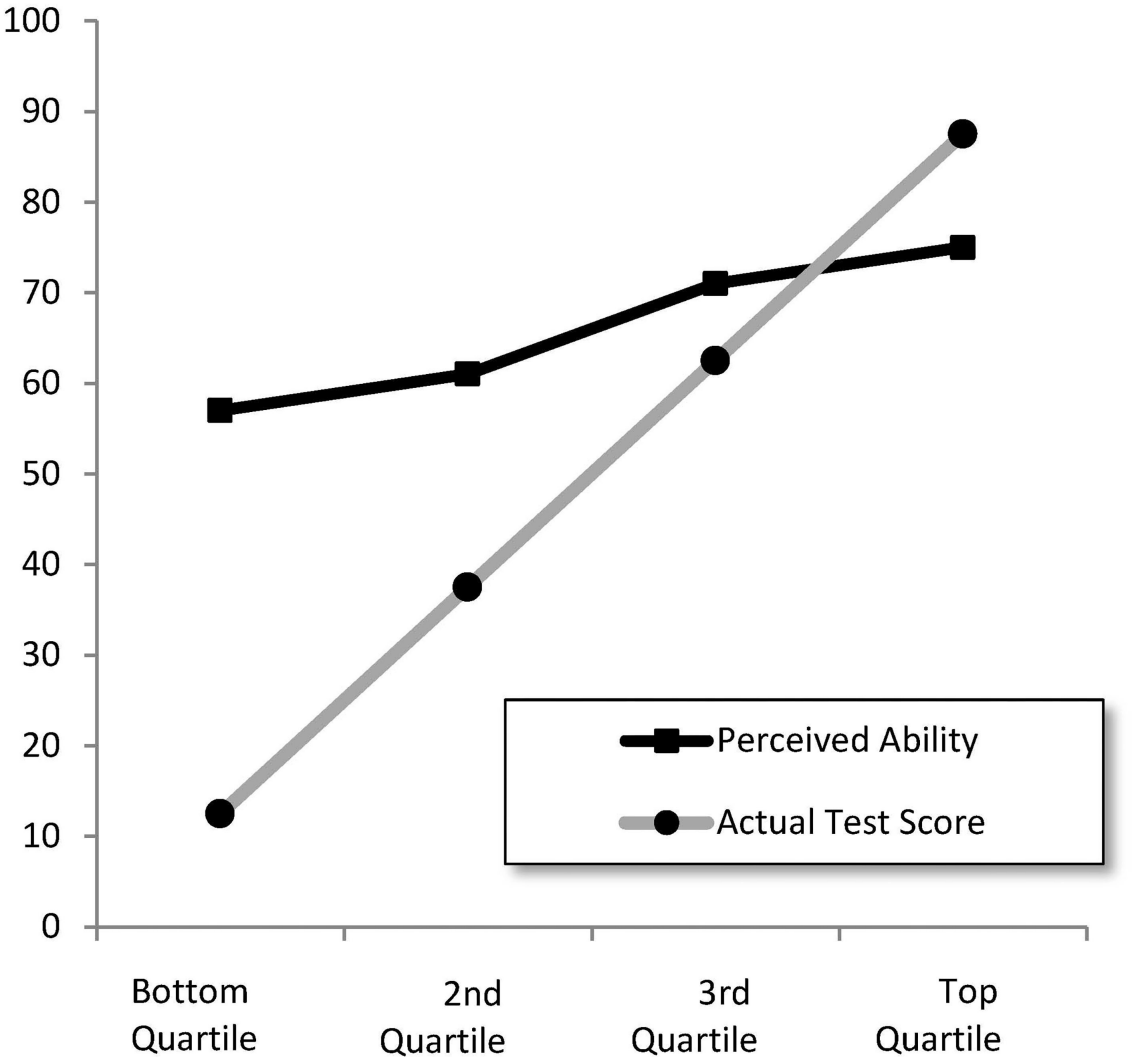
Perceived ability to recognize humor as a function of actual test performance (from Kruger and Dunning, 1999).

In their original article, Kruger and Dunning (1999) tested undergraduate students enrolled in various psychology courses at Cornell University for their ability in humor, logical reasoning, and English grammar. After the test they asked the students to assess their performance in the test. The students were then split in four groups according to their actual test scores. Calculating the average perceived ability in each group, Dunning and Kruger obtained Figure 1. The accuracy of the prediction was high in the top group and low in the bottom group, and the prediction in the bottom group was highly overestimated.

The Kruger–Dunning article raises two questions. First, is there a DK effect? And second, is the explanation provided by Dunning and Kruger correct?

There has been both criticism and support. Most studies recognize that there is a DK effect and provide a psychological explanation, sometimes agreeing, sometimes disagreeing with Kruger and Dunning's metacognitive explanations; see Ehrlinger et al. (2008), Schlosser et al. (2013), Williams et al. (2013), Sullivan et al. (2018), West and Eaton (2019), Gabbard and Romanelli (2021), and Mariana et al. (2021); and partial responses in Kruger and Dunning (2002), Dunning et al. (2003, 2004), and Dunning (2011).

Meeran et al. (2016) explained the DK effect based on the anchoring and adjustment heuristic, while Jansen et al. (2021) replicated two of Dunning and Kruger's studies using a sample of 4,000 participants. Their model for the probability of a correct answer is an extension of the one-parameter item response theory (IRT) model, known as the Rasch model (Embretson and Reise, 2013). By developing a rational model of self-assessment, they showed that the DK effect can be produced by two psychological mechanisms.

But there has also been much criticism and this criticism typically relies on a statistical rather than a psychological explanation of the DK effect. The attack on Dunning and Kruger was initiated by Krueger and Mueller (2002), who suggested a regression better-than-average approach which is parsimonious and “does not require mediation by third variables, such as metacognitive insights into one's own problem-solving abilities^1^.” Their approach is based on two empirical facts. First, it is well-known that people tend to overestimate their performance. Most people think they drive better than average (Svenson, 1981). In a survey of engineers, 42% thought their work ranked in the top 5% among their peers (Zenger, 1992); and in a survey of college professors, 94% thought they performed “above average” (Cross, 1977). Second, the slope in the linear regression of estimated performance on actual performance is not equal but less than one. This phenomenon is called “regression to the mean” and has been known since Galton (1886) studied the relationship between the height of sons and fathers. Combining these two facts leads to the regression better-than-average approach, and it explains the asymmetry of the DK effect: overestimation in the bottom quartile and underestimation in the upper quartile. A more precise formulation of the regression better-than-average approach was provided in the noise-plus-bias model (Burson et al., 2006).

The DK effect also occurs in different environments, for example the problem of face recognition. This situation was recently investigated by Kramer et al. (2022), who found imperfect correlation between self-insight and competence.

Several studies attempted to provide statistical explanations. Ackerman et al. (2002) demonstrated that pictures like Figure 1 can be obtained with simulated data using two random variables with small correlation *r* = 0.19 (one representing objective knowledge and the other self-reported knowledge). Ackerman and Wolman (2007) studied post-test self-estimates of “Raven” performance, using a scatter plot with a regression line representing the effect of self-estimated Raven performance on Raven's actual performance. They found a correlation less than 1 and also the slope of the regression line was less than 1, demonstrating the DK effect. Nuhfer et al. (2016) used simulated random variables with correlation less then 1 and various graphical representations of the data to illustrate the DK effect. Krajc and Ortmann (2008) assumed a nonsymmetric *J*-distribution for the talent of the undergraduates studied by Kruger and Dunning (1999), which leads to more students in the left tail of the students' ability distribution, resulting in the DK effect. McIntosh et al. (2019) experimented with movement and memory tasks, and concluded that the DK effect exists as an empirical phenomenon. But they disagreed with the explanation that poor insight is the reason for overestimation among the unskilled. Gignac and Zajenkowski (2020), using a sample of general community participants, tested the validity of the DK effect with the Glejser test of heteroskedasticity and by nonlinear (quadratic) regression, and found much less evidence in favor of the DK effect than Kruger and Dunning (1999).

Our explanation of the DK effect is based on the fact that the data are bounded. This feature of the data has not received much attention, with the exception of Burson et al. (2006), who concluded that the boundary restriction “is an important concern that should be addressed in future research;” and Krajc and Ortmann (2008), who noted that students in the bottom quartile can only make optimistic errors placing themselves into a higher quartile, while students in the top quartile can only make pessimistic errors placing themselves in a lower quartile.

The remark by Krajc and Ortmann provides the essence of our story. Consider a brilliant student who typically scores 95 or 99 points out of 100. Because of the bound at 100, there is not much room to predict higher than her ability but there is plenty of room to predict lower, so she would typically predict 85 or 90, thus underestimating her score. The same happens at the bottom end of the scale, where there is a bound of 0 and a student would typically overestimate. This simple observation is the basis of our model.

We shall employ data on 665 undergraduates at the International College of Economics and Finance of the Higher School of Economics in Moscow, who predict their grade on a 0–100 scale for a statistics exam. We use a simple statistical model which explicitly specifies the (random) boundary constraints. This model fits the data almost perfectly. There is thus no need for a psychological explanation of the DK effect: it is a statistical artifact.

The remainder of this article is organized as follows. First, we present and discuss the data. Next, we present a simple one-parameter model that accounts for censuring, and show that this model explains the DK effect, although not yet perfectly. Then, we extend this simple model to a more realistic three-parameter model where the bounds are random rather than fixed, and present the results based on this extended model. The fit is now near-perfect. The final section concludes and provides a tentative explanation of why it is that people still believe the psychological underpinnings of the DK effect. A mathematical Appendix contains the statistical theory underlying the required conditional expectation functions.

## The Data

We shall study the DK effect by comparing exam results with predictions of these results, and, in this section, we describe the data in some detail.

The International College of Economics and Finance (ICEF) in Moscow was established in 1997 jointly by the London School of Economics and Political Science (LSE) in London and the Higher School of Economics (HSE) in Moscow. The college offers a four-year bachelor's program, which is considered to be one the top programs in economics in Russia. Each year about 200 students enter the program, typically immediately after high school. In their first year the students follow, among other subjects, a course called Statistics-1, and in their second year they follow Statistics-2. Both courses are compulsory. Our data are obtained from four cohorts of students following Statistics-2 in the period 2016–2019. In total, after removing students who took the course for a second time, 665 students remained who took this course and provided a prediction.

In Statistics-2 students take three exams every year, at the end of October (exam 1), the end of December (exam 2), and the end of March (exam 3). The exams are written exams, not multiple choice, and each exam consists of two parts (80 min each) with a ten min break between the two parts. The level of the exam questions is the same in the two parts. To avoid cheating, students are not allowed to leave and come back during each part of the exam. At the end of part 1 and at the end of part 2 the examiner collects each student's work. Each part is graded out of 50 points.

At the end of the first part of each of the three exams each student is invited to predict (out of 100) their grade for this exam (the two parts together). When writing down the prediction, students know the questions and their answers in part 1, but not yet the questions of part 2. To encourage students to provide a prediction and try their best, a bonus is promised as follows. If the difference between the prediction and the grade is less than or equal to 3 in absolute value, then one bonus point is added to the grade. For example, if the prediction is 49 and the grade is 52, then the grade for this exam is marked up to 53. This procedure had to be and has been approved by the ICEF administration. As a result of the procedure and the possibility of a bonus, the response rate was extremely high (97%). The idea of giving each student an incentive to express their opinion was successfully used earlier in experiments by Blackwell (2010) and Magnus and Peresetsky (2018). Each of the two parts typically consists of four problems (not multiple choice). Each problem was graded by the same class teacher, thus improving the objectivity of the grades (see Meeran et al., 2016).

In the current study, we take data only from the second exam in each year. This is the most representative of the three exams, because in the first exam students may not yet be familiar with the benefits of a careful prediction, and in the third exam there is the problem that smart (or risk averse) students utilize the bonus to maximize the probability that their grade is ≥25, which is a requirement for passing the course. The student's optimal strategy is then to choose their prediction between 21 and 27 in which case a grade of 24 would be marked up to 25. Many students actually use this strategy which leads to an overrepresentation of 24 and 25 in the sample of the third exam.

In each year *t* we thus have one grade and one prediction per student. Let us define


$$\begin{array}{l} \;x_{it}:\text{actual grade of student}i\text{in year}t,\\ \;y_{it}^{raw}:\text{raw (unadjusted) prediction of student}\\ \;i\text{ in year}t,\\ d_{it}=y_{it}^{raw}-x_{it}:\text{difference between raw prediction and}\\ \;\text{actual grade.} \end{array}$$

In each year we can average over students and this gives


$${\bar{x}}_{t}=\frac{1}{n_{t}}\overset{n_{t}}{\underset{i=1}{\sum }}x_{it},\;{ȳ}_{t}^{raw}=\frac{1}{n_{t}}\overset{n_{t}}{\underset{i=1}{\sum }}y_{it}^{raw},\;{\bar{d}}_{t}=\frac{1}{n_{t}}\overset{n_{t}}{\underset{i=1}{\sum }}d_{it},$$

where *n*_*t*_ denotes the number of students in year *t*.

We don't want to use the raw predictions directly, because of the variation in the student cohort's strengths and in the difficulty of the exam over the years. To filter out these variations we define an adjusted prediction


(1) $$\begin{array}{r} y_{it}=y_{it}^{raw}-{\bar{d}}_{t} \end{array}$$

with the property that ${ȳ}_{t}={\bar{x}}_{t}$, so that in each year the average prediction equals the average grade.

In Table 1, we present a summary of the data. Per year we provide the number of students *n*_*t*_, the average exam grade ${\bar{x}}_{t}$, the average raw prediction ${ȳ}_{t}^{raw}$, and the difference ${\bar{d}}_{t}$ between these two averages. The difference *d*_*it*_ varies a lot within each year, as shown by the standard deviation τ_*t*_ in the last column. The second exam in 2017 turned out particularly difficult (or the cohort was less motivated) leading to relatively low grades.

**Table 1:** Descriptive statistics (means) of the data.

**Year**	**# Students**	**Exam grade**	**Raw prediction**	**Difference**	**St. dev**.
**t**	***n*_*t*_**	${\bar{x}}_{t}$	${ȳ}_{t}^{raw}$	${\bar{d}}_{t}$	**τ_*t*_**
2016	144	41.8	39.0	−2.76	12.1
2017	168	33.3	38.5	5.24	12.7
2018	185	41.2	37.5	−3.71	13.1
2019	168	43.0	39.3	−3.65	12.0
Total	665	39.8	38.6	−1.23	13.0

## Fixed Bounds

As a first attempt to model the predictions we propose the following equation:


(2) $$\begin{array}{r} y_{it}^{raw}=\alpha_{t}+x_{it}+\zeta_{it}, \end{array}$$

where the constant α_*t*_ may vary per year to adjust for (over)confidence and the difficulty of the exam, and the errors ζ_*it*_ are assumed to be independent and identically distributed as $\text{N}\left(0,\sigma_{\zeta }^{2}\right)$. Writing Equation (2) in deviation form gives


$$y_{it}^{raw}-{ȳ}_{t}^{raw}=\left(x_{it}-{\bar{x}}_{t}\right)+\left(\zeta_{it}-{\bar{\zeta }}_{t}\right).$$

The adjusted prediction is given by


$$y_{it}=y_{it}^{raw}-{\bar{d}}_{t}=y_{it}^{raw}-{ȳ}_{t}^{raw}+{\bar{x}}_{t},$$

which leads to the simple equation


$$\begin{array}{l} y_{it}=x_{it}+\epsilon_{it},\;\epsilon_{it}=\zeta_{it}-{\bar{\zeta }}_{t}. \end{array}$$

After adjustment, the year plays no longer any role, so we may simplify the notation and write


(3) $$\begin{array}{r} y_{i}=x_{i}+\epsilon_{i}. \end{array}$$

The difference between the (adjusted) prediction *y*_*i*_ and the grade *x*_*i*_ is thus random noise, and the only thing to estimate is the variance of that noise.

This first attempt does not, however, take into account that the left-hand side of Equation (3) is bounded by 0 ≤ *y*_*i*_ ≤ 100, so that the right-hand side is similarly bounded. The right-hand side *x*_*i*_ + ϵ_*i*_ does not automatically fulfill this constraint; it has to be censored to do so. The basic censuring model in statistics and econometrics is the tobit model introduced by Tobin (1958). In the tobit model, we introduce a latent (unobserved) random variable $y_{i}^{*}$ defined as


(4) $$\begin{array}{r} y_{i}^{*}=x_{i}+\epsilon_{i}, \end{array}$$

where the ϵ_*i*_ are independent and identically distributed as $\text{N}\left(0,\sigma_{\epsilon }^{2}\right)$. Then, we model *y*_*i*_ as


(5) $$y_{i}=\{\begin{array}{ll} 0, & \text{ if }y_{i}^{*}\leq 0,\\ y_{i}^{*}, & \text{ if }0<y_{i}^{*}\leq 100,\\ 100, & \text{ if }y_{i}^{*}>100. \end{array}$$

This is the standard tobit model, double-censured due to the fact that we have both a lower and an upper bound.

Model Equation (5) is more realistic than model Equation (3), and once we have estimated $\sigma_{\epsilon }^{2}$ we can compute the expectation *h*(*x*_*i*_) = E(*y*_*i*_) as given in Equation (9) in the Appendix.

**Figure 2:**
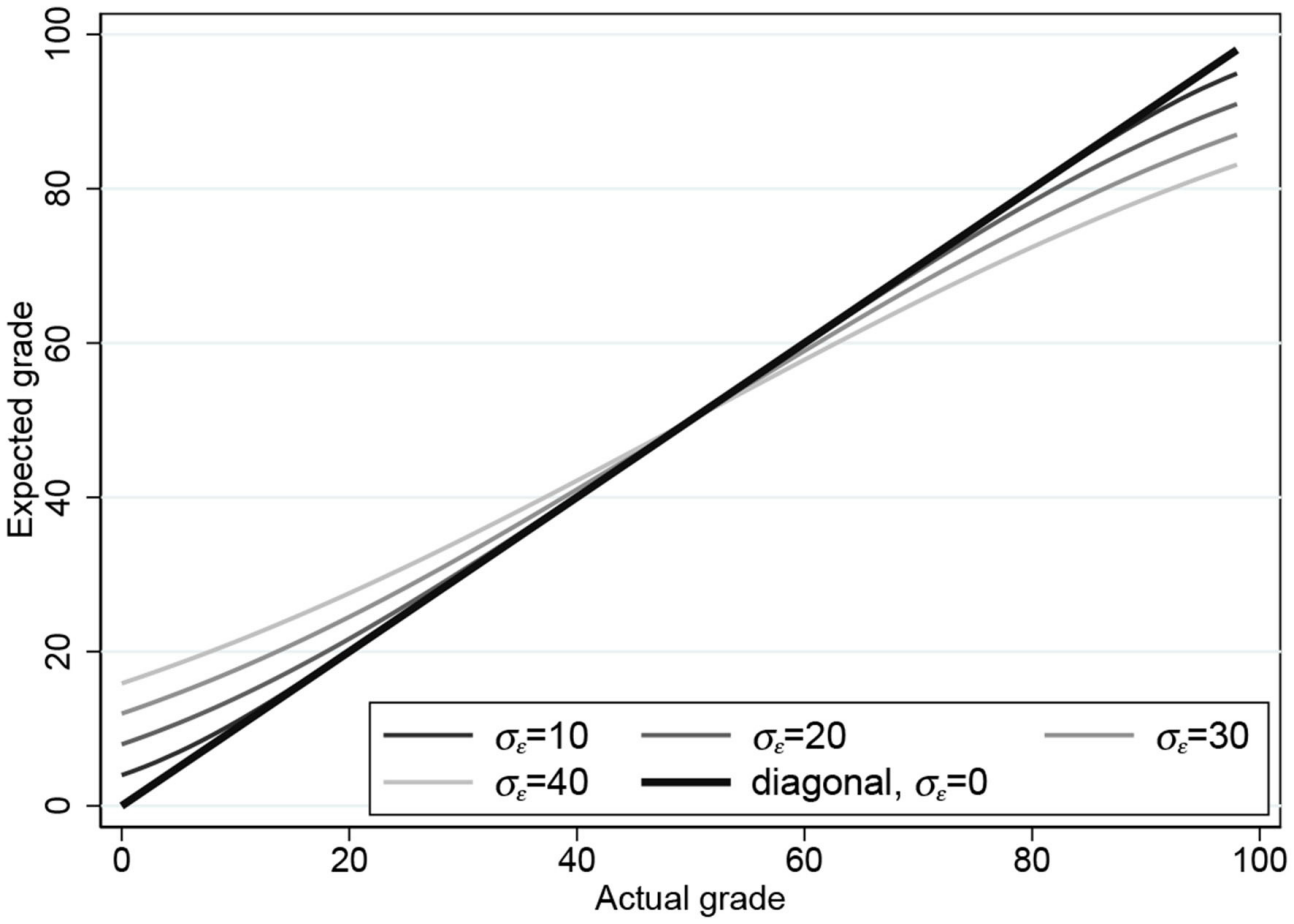
Expectation functions for the one-parameter censored tobit model for σ_ϵ_ = 0, 10, 20, 30, 40.

Estimating σ_ϵ_ by maximum likelihood (ML) gives ${\hat{\sigma }}_{\epsilon }=12.5$ with standard error 0.35. In Figure 2, we plot the expectation *h*(*x*_*i*_) = E(*y*_*i*_) for four values of σ_ϵ_: 10, 20, 30, and 40 (and 0, which is the 45° line). Figure 2 already demonstrates how the two bounds force the expectation function in the direction of the DK effect in Figure 1.

But the fixed-bound model is not yet completely satisfactory due to the fact that it is not realistic to assume that *y*_*i*_ = 0 if $y_{i}^{*}\leq 0$ or that *y*_*i*_ = 100 if $y_{i}^{*}>100$: no student predicts 0 (however, poor) or 100 (however, brilliant). This leads to the random-bounds model presented in the next section.

## Random Bounds

A more realistic model is given by


(6) $$y_{i}=\{\begin{array}{ll} |u_{i}|, & \text{ if }y^{*}\leq 0,\\ y_{i}^{*}, & \text{ if }0<y^{*}\leq 100,\\ 100-|v_{i}|, & \text{ if }y^{*}>100, \end{array}$$

where we assume that ϵ_*i*_, *u*_*i*_, and *v*_*i*_ are independent and identically distributed, that they are independent of each other, and that all three are normally distributed as $\text{N}\left(0,\sigma_{\epsilon }^{2}\right)$, $\text{N}\left(0,\sigma_{u}^{2}\right)$, and $\text{N}\left(0,\sigma_{v}^{2}\right)$, respectively.

When applying Equation (6) there is one further complication, namely that the lower bound must not only satisfy |*u*_*i*_| > 0, but also |*u*_*i*_| < 100, while the upper bound must not only satisfy 100 − |*v*_*i*_| < 100, but also 100−|*v*_*i*_| > 0. Hence, we must require that |*u*_*i*_| <100 and |*v*_*i*_| < 100. This will be “almost” true in most applications. For example, we have Pr(|*u*_*i*_| < 100) = 99.9 and 95.5 for σ_*u*_ = 30 and 50, respectively. We deal formally with this situation by considering the conditional expectation function


(7) $$\begin{array}{r} h\left(x_{i}\right)=E\left(y_{i}|0<y_{i}<100\right). \end{array}$$

We derive the mathematical expression for this conditional expectation function in the Appendix, resulting in Equation (8).

The ML estimates are presented in Table 2, first without restriction and then under the restriction that σ_*u*_ = σ_*v*_. The estimates take on reasonable values and they are estimated rather precisely. The restriction σ_*u*_ = σ_*v*_ is not rejected by a Wald (*p*-value 0.061) or likelihood ratio (*p*-value 0.412) test.

**Table 2:** Maximum likelihood estimates for the three-parameter model (standard errors in parentheses).

**Restriction**	**σ_ϵ_**	**σ_*u*_**	**σ_*v*_**	**Log L**
None	12.69	22.81	31.29	−2582.39
	(0.40)	(2.81)	(3.38)	
σ_*u*_ = σ_*v*_	12.67	24.79	24.79	−2582.73
	(0.39)	(2.30)	(2.30)	

**Figure 3:**
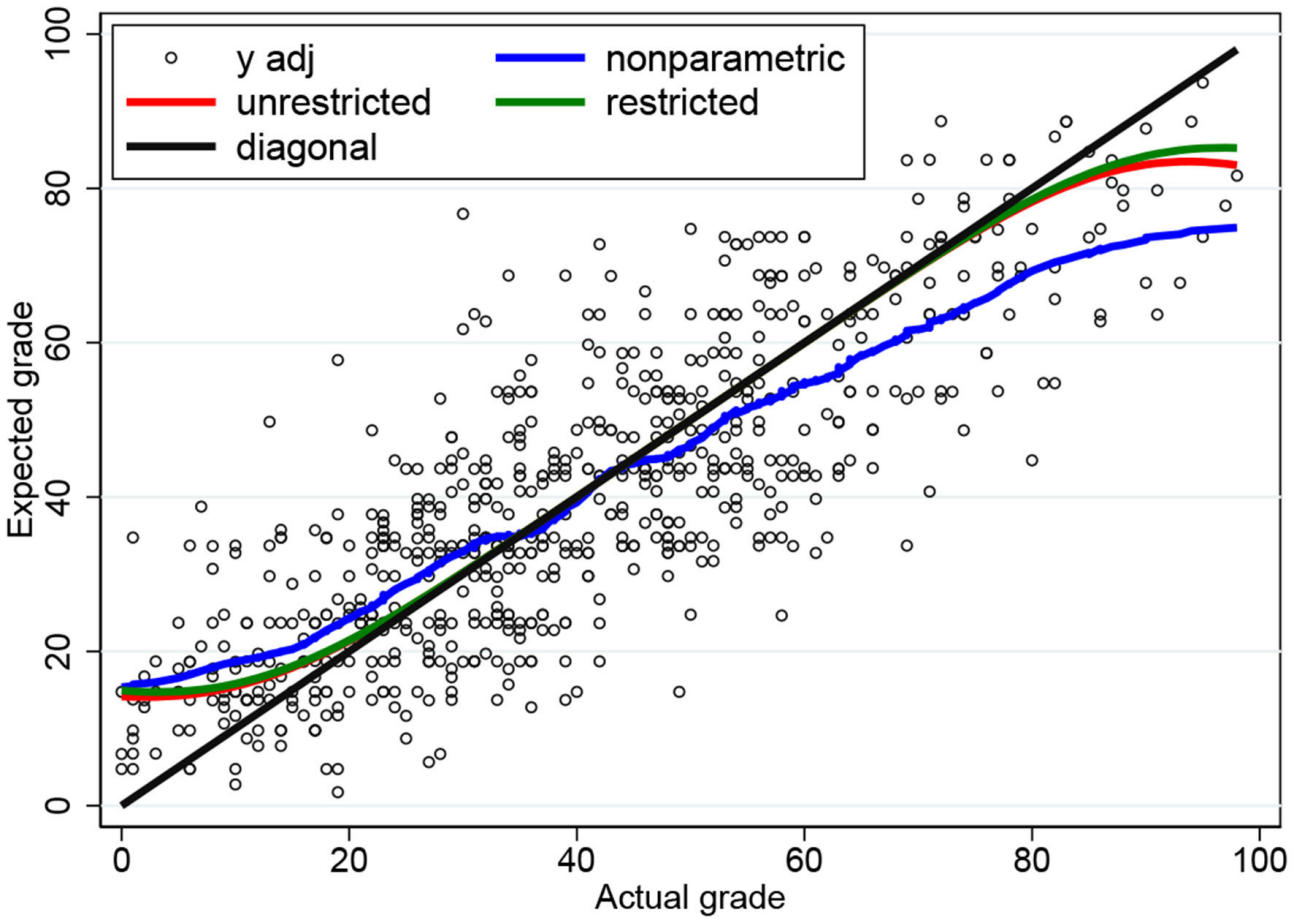
Conditional expectation functions for the three-parameter censored tobit model based on ML and nonparametric estimates.

In Figure 3, we present the conditional expectation functions based on the ML estimates in Table 2. As expected, there is not much difference between the restricted (σ_*u*_ = σ_*v*_) and the unrestricted plot, and both plots clearly show the DK effect based purely on the fact that the observations are bounded. The observed *S*-shape is very similar to the empirical plots reported in the literature: overestimation for the weak students (“unskilled and unaware of it” in the words of Dunning and Kruger) and underestimation for the strong students.

As a benchmark comparison we also provide a nonparametric plot based on locally-weighted scatter plot smoothing (lowess) with bandwidth 0.2. The ML plots are close to the nonparametric plot when *x* is small but not so close when *x* is large. This is caused by the fact that only 14% of the observations fall in the interval *x*>60.

**Figure 4:**
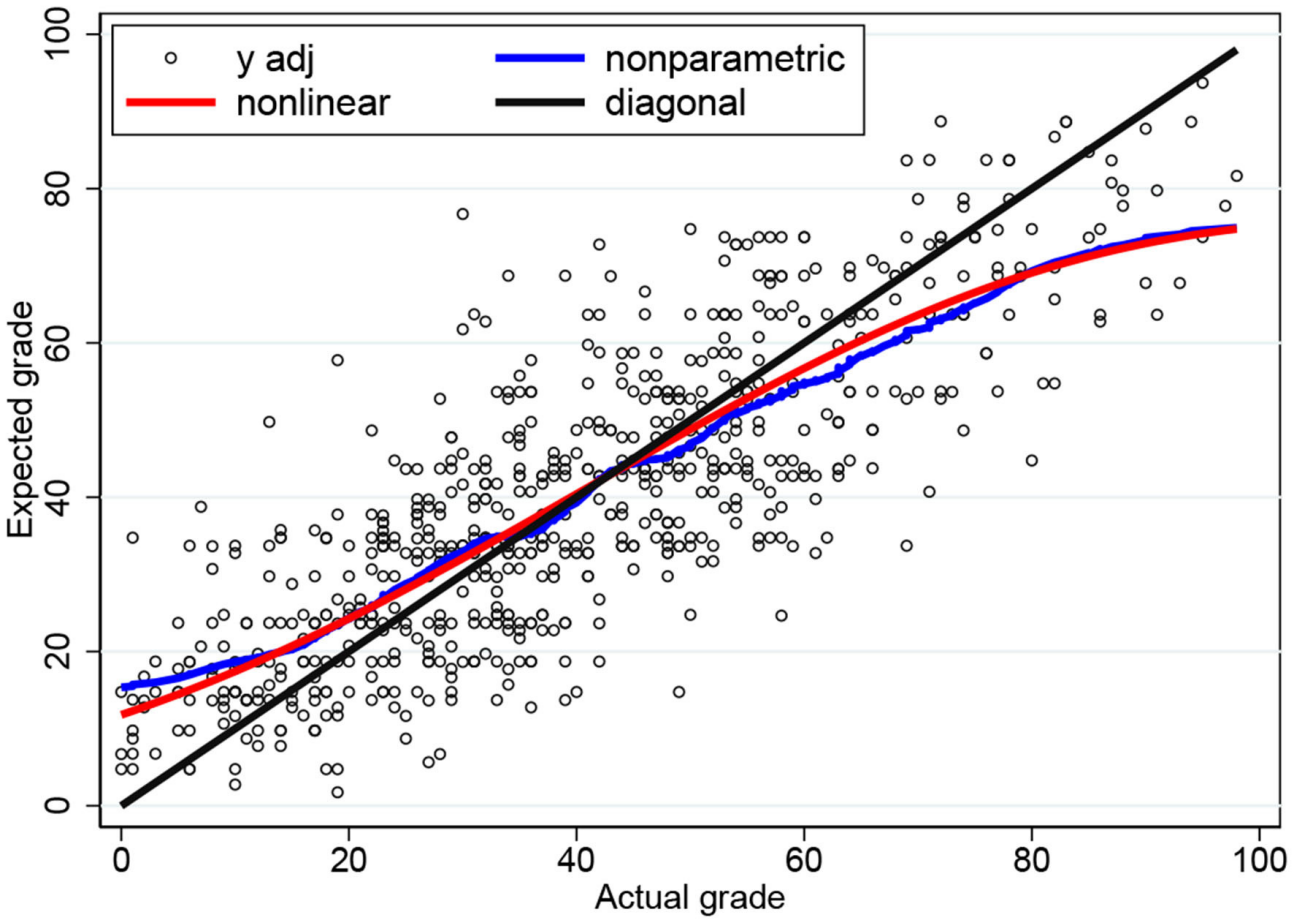
Conditional expectation functions for the three-parameter censored tobit model based on NLS and nonparametric estimates.

We can try and fit our three parameters such that the conditional expectation function is “as close as possible” to the nonparametric plot. If we employ nonlinear least squares (NLS), then we find ${\hat{\sigma }}_{\epsilon }=29.6\left(0.70\right)$, ${\hat{\sigma }}_{u}=0$, and ${\hat{\sigma }}_{v}=33.1\left(2.13\right)$. The NLS plot in Figure 4 is now very close to the nonparametric plot. In fact, the shape of the conditional expectation function is quite robust against changes in the three parameters. If we fix the parameters at σ_ϵ_ = 30, σ_*u*_ = 10, and σ_*v*_ = 35; or at σ_ϵ_ = 25, σ_*u*_ = 15, and σ_*v*_ = 40, then we obtain conditional expectations that are almost indistinguishable from Figure 4.

## Discussion and Conclusions

In this article, we have attempted to provide an explanation of the DK effect which does not require any psychological explanation. By specifying a simple statistical model which explicitly takes the (random) boundary constraints into account, we achieve a near-perfect fit, thus demonstrating that the DK effect is a statistical artifact. In other words: there is an effect, but it does not reflect human nature.

Many authors writing on the DK effect refer to regression to the mean (RM) as if this were a well-known statistical fact, not requiring further justification, while in fact the mechanism underlying the RM effect needs to be explained in each application. A “psychological” explanation of the RM effect was suggested by Wellman (1940), though instantly criticized by Goodenough and Maurer (1940).

Before we discuss the RM effect further, let's point out a common confusion in its interpretation. Suppose we have data on two variables *x* and *y*, say (*x*_1_, *y*_1_), …, (*x*_*n*_, *y*_*n*_). Then we can compute the sample correlation *r*_*xy*_. We can also regress *y* on *x*, estimating α and β from the regression *y*_*i*_ = α+β*x*_*i*_ + ϵ_*i*_. The least-squares estimator of β can be written as $\hat{\beta }=r_{xy}s_{y}/s_{x}$, where $s_{x}^{2}$ and $s_{y}^{2}$ are the sample variances of *x* and *y*, respectively. The term “imperfect correlation” is used to indicate that 0 < *r*_*xy*_ < 1. Regression to the mean on the other hand is equivalent to $0<\hat{\beta }<1$. Confusion arises because the two conditions are not the same. When *r*_*xy*_ = 1 and *s*_*y*_ < *s*_*x*_, then $\hat{\beta }<1$ so that there is regression to the mean in spite of the fact that *r*_*xy*_ = 1. Vice versa, when $\hat{\beta }=1$ and *s*_*y*_ > *s*_*x*_, then *r* < 1 so that there is no regression to the mean in spite of the fact that *r*_*xy*_ < 1.

The RM effect was first described by sir Francis Galton in 1886. Galton compared the heights of parents and children and found that if the parents are tall, then the children are, on average, shorter than their parents, and that if the parents are short, the children are, on average, taller. He explained the RM effect by assuming that there are two genes responsible for height: one is inherited from the parents, and the other reflects the average height in the population. The height of the children can then be represented as a weighted average of the two.

Galton's explanation was appealing and brilliant, given the knowledge on genetics at that time. Today we know that height is determined by about 700 genes in approximately 180 locations in the chromosomes (Allen et al., 2010), and the RM effect can be explained in a probabilistic framework from the fact that of each pair of chromosomes one is inherited from the mother and one from the father.

But there could be bounds here too, not “hard” bounds (like 0 and 100 in exams) but “soft” bounds (say, 130 and 230 cm). Suppose the inherited height equals the average height of the parents with some random shock. If the inherited height is too high or too low then the probability of prenatal death is high, which explains (at least in part) the RM effect as a statistical artifact caused by these bounds. In fact, our presented theory based on boundaries can explain the RM effect in many areas, not just students' grades or children's heights.

Finally, why do people still believe in psychological explanations of the DK effect? The literature abounds with characters who overestimate themselves and with wisdoms about how stupid it is to think you are clever. Shakespeare writes: “The fool doth think he is wise, but the wise man knows himself to be a fool” (*As You Like It*), and Alexander Pope warns: “A little learning is a dangerous thing” (*An Essay on Criticism*), in the same spirit as Confucius' maxim: “Real knowledge is to know the extent of one's ignorance.”

Perhaps the explanation for the persistence of this belief is: We have two facts, both true. First, we actually observe the DK effect. Second, if we compare people's ideas about their own ability with objective measurements of this ability, we find that people tend to overestimate themselves. Then, what is more natural than to think that these two statements are related to each other, in fact, that one causes the other? The problem is, they aren't.

## Data Availability Statement

The original contributions presented in the study are included in the article/supplementary material, further inquiries can be directed to the corresponding author/s.

## Ethics Statement

Ethical review and approval was not required for the study on human participants in accordance with the local legislation and institutional requirements. Written informed consent from the patients/ participants or patients/participants legal guardian/next of kin was not required to participate in this study in accordance with the national legislation and the institutional requirements.

## Author Contributions

All authors listed have made a substantial, direct, and intellectual contribution to the work and approved it for publication.

## Conflict of Interest

The authors declare that the research was conducted in the absence of any commercial or financial relationships that could be construed as a potential conflict of interest.

## Publisher's Note

All claims expressed in this article are solely those of the authors and do not necessarily represent those of their affiliated organizations, or those of the publisher, the editors and the reviewers. Any product that may be evaluated in this article, or claim that may be made by its manufacturer, is not guaranteed or endorsed by the publisher.

## Footnotes

^1^To avoid confusion: Joachim Krueger of Brown University and Justin Kruger of Cornell University are two different people.

## Mathematical Appendix

Consider the censored model Equation (6), where we write *y*^*^, *y*, and *x* instead of $y_{i}^{*}$, *y*_*i*_, and *x*_*i*_ for simplicity. We have

$$\begin{array}{l} p_{1}=\mathrm{Pr}\left(y^{*}\leq 0\right)=\Phi \left(\frac{-x}{\sigma_{\epsilon }}\right),\\ p_{2}=\mathrm{Pr}\left(0<y^{*}\leq 100\right)=1-p_{1}-p_{3},\\ p_{3}=\mathrm{Pr}\left(y^{*}>100\right)=1-\Phi \left(\frac{100-x}{\sigma_{\epsilon }}\right), \end{array}$$

where Φ denotes the cumulative distribution function of the standard-normal distribution, and

$$\begin{array}{l} q_{1}=\mathrm{Pr}\left(y\leq 0\right)=\mathrm{Pr}\left(y\leq 0|y^{*}>100\right)\cdot \mathrm{Pr}\left(y^{*}>100\right)\\ \;=\mathrm{Pr}\left(100-\sigma_{v}|v|\leq 0\right)\cdot p_{3}=2p_{3}\Phi \left(\frac{-100}{\sigma_{v}}\right),\\ q_{2}=\mathrm{Pr}\left(0<y\leq 100\right)=1-q_{1}-q_{3},\\ q_{3}=\mathrm{Pr}\left(y>100\right)=\mathrm{Pr}\left(y>100|y^{*}\leq 0\right)\cdot \mathrm{Pr}\left(y^{*}\leq 0\right)\\ \;=\mathrm{Pr}\left(\sigma_{u}|u|>100\right)\cdot p_{1}=2p_{1}\Phi \left(\frac{-100}{\sigma_{u}}\right). \end{array}$$

Let 0 < *t* < 100. Then,

$$\begin{array}{l} G_{1}\left(t\right)=\mathrm{Pr}\left(0<y<t|y^{*}\leq 0\right)=\mathrm{Pr}\left(0<\sigma_{u}|u|<t\right)\\ \;=1-2\Phi \left(\frac{-t}{\sigma_{u}}\right),\\ G_{2}\left(t\right)=\mathrm{Pr}\left(0<y<t|0<y^{*}\leq 100\right)\\ \;=\mathrm{Pr}\left(0<y^{*}<t|0<y^{*}\leq 100\right)\\ \;=\frac{\mathrm{Pr}\left(0<y^{*}<t\right)}{\mathrm{Pr}\left(0<y^{*}<100\right)}=\frac{\mathrm{Pr}\left(0<x+\sigma_{\epsilon }\epsilon <t\right)}{p_{2}}\\ \;=\frac{1}{p_{2}}\left[\Phi \left(\frac{t-x}{\sigma_{\epsilon }}\right)-\Phi \left(\frac{-x}{\sigma_{\epsilon }}\right)\right],\\ G_{3}\left(t\right)=\mathrm{Pr}\left(0<y<t|y^{*}>100\right)=\mathrm{Pr}\left(0<100-\sigma_{v}|v|<t\right)\\ \;=2\left[\Phi \left(\frac{100}{\sigma_{v}}\right)-\Phi \left(\frac{100-t}{\sigma_{v}}\right)\right] \end{array}$$

with derivatives

$$\begin{array}{l} g_{1}\left(t\right)=\frac{2}{\sigma_{u}}\phi \left(\frac{-t}{\sigma_{u}}\right),\\ g_{2}\left(t\right)=\frac{1}{p_{2}\sigma_{\epsilon }}\phi \left(\frac{t-x}{\sigma_{\epsilon }}\right),\\ g_{3}\left(t\right)=\frac{2}{\sigma_{v}}\phi \left(\frac{100-t}{\sigma_{v}}\right), \end{array}$$

where ϕ = Φ′ denotes the density of the standard-normal distribution. This gives

$$\begin{array}{l} m_{1}=\int_{0}^{100}tg_{1}\left(t\right)dt=2\sigma_{u}\int_{0}^{100/\sigma_{u}}s\phi \left(s\right)ds\\ \;=-2\sigma_{u}\int_{0}^{100/\sigma_{u}}\phi^{\prime }\left(s\right)ds=2\sigma_{u}\left(\phi \left(0\right)-\phi \left(\frac{100}{\sigma_{u}}\right)\right),\\ m_{2}=\int_{0}^{100}tg_{2}\left(t\right)dt=\frac{x}{p_{2}}\int_{-x/\sigma_{\epsilon }}^{\left(100-x\right)/\sigma_{\epsilon }}\phi \left(s\right)ds\\ \;+\frac{\sigma_{\epsilon }}{p_{2}}\int_{-x/\sigma_{\epsilon }}^{\left(100-x\right)/\sigma_{\epsilon }}s\phi \left(s\right)ds\\ \;=\frac{x}{p_{2}}\left(\Phi \left(\frac{100-x}{\sigma_{\epsilon }}\right)-\Phi \left(\frac{-x}{\sigma_{\epsilon }}\right)\right)\\ \;-\frac{\sigma_{\epsilon }}{p_{2}}\left(\phi \left(\frac{100-x}{\sigma_{\epsilon }}\right)-\phi \left(\frac{-x}{\sigma_{\epsilon }}\right)\right),\\ m_{3}=\int_{0}^{100}tg_{3}\left(t\right)dt=200\int_{-100/\sigma_{v}}^{0}\phi \left(s\right)ds\\ \;+2\sigma_{v}\int_{-100/\sigma_{v}}^{0}s\phi \left(s\right)ds\\ \;=200\left(\Phi \left(0\right)-\Phi \left(\frac{-100}{\sigma_{v}}\right)\right)-2\sigma_{v}\left(\phi \left(0\right)-\phi \left(\frac{-100}{\sigma_{v}}\right)\right). \end{array}$$

Letting

$$\begin{array}{l} F\left(t\right)=\mathrm{Pr}\left(0<y<t|0<y<100\right)\\ \;=\frac{p_{1}G_{1}\left(t\right)+p_{2}G_{2}\left(t\right)+p_{3}G_{3}\left(t\right)}{q_{2}} \end{array}$$

with derivative

$$f\left(t\right)=\frac{p_{1}g_{1}\left(t\right)+p_{2}g_{2}\left(t\right)+p_{3}g_{3}\left(t\right)}{q_{2}},$$

we thus obtain

(8)
$$\begin{array}{r} \int_{0}^{100}tf\left(t\right)dt=\frac{p_{1}m_{1}+p_{2}m_{2}+p_{3}m_{3}}{q_{2}}, \end{array}$$

which provides the expectation in the general case.

In the special case of model Equation (5) we have σ_*u*_ = σ_*v*_ = 0, and hence *m*_1_ = 0, *m*_3_ = 100, and *q*_2_ = 1. This gives the expectation

(9)
$$\begin{array}{r} \int_{0}^{100}tf\left(t\right)dt=p_{2}m_{2}+100p_{3}. \end{array}$$
